# Computational Genomics of Specialized Metabolism: from Natural Product Discovery to Microbiome Ecology

**DOI:** 10.1128/mSystems.00182-17

**Published:** 2018-03-06

**Authors:** Marnix H. Medema

**Affiliations:** a Bioinformatics Group, Wageningen University, Wageningen, The Netherlands

**Keywords:** bioinformatics, biosynthetic gene cluster, microbiome, natural products, specialized metabolism

## Abstract

Microbial and plant specialized metabolites, also known as natural products, are key mediators of microbe-microbe and host-microbe interactions and constitute a rich resource for drug development. In the past decade, genome mining has emerged as a prominent strategy for natural product discovery. Initially, such mining was performed on the basis of individual microbial genome sequences. Now, these efforts are being scaled up to fully genome-sequenced strain collections, pangenomes of bacterial genera, and large sets of metagenome-assembled genomes from microbial communities. The Medema research group aims to play a leading role in these developments by developing and applying computational approaches to identify, classify, and prioritize specialized metabolite biosynthetic gene clusters and pathways and to connect them to specific molecules and microbiome-associated phenotypes. Moreover, we are extending the scope of genome mining from microbes to plants, which will allow more comprehensive interpretation of the chemical language between hosts and microbes in a microbiome setting.

## Abstract (precis)

Microbial and plant specialized metabolites, also known as natural products, are key mediators of microbe-microbe and host-microbe interactions and constitute a rich resource for drug development. In the past decade, genome mining has emerged as a prominent strategy for natural product discovery.

## PERSPECTIVE

Bacteria, fungi, and plants produce a wide range of specialized metabolites (also known as natural products) that allow them to thrive in their environments. In microbiomes, these molecules play key roles in competition and collaboration by serving as signals, weapons, nutrient-scavenging agents, and stress protectants. Many different chemical classes of natural products exist, including terpenes, polyketides, peptides, saccharides, and alkaloids. Thousands of these molecules are applied in human society as crop protection agents, antibiotics, chemotherapeutics, immunosuppressants, surfactants, and ingredients for food manufacturing.

The genes encoding natural product biosynthetic pathways are frequently physically clustered on the chromosome of the producing organism. Over 1,500 of these biosynthetic gene clusters BGCs) and their products have now been characterized experimentally (1). Intriguingly, this physical clustering makes it straightforward to identify biosynthetic pathways for novel molecules through computational genomic analysis, regardless of the fact that many BGCs are transcriptionally silent under typical laboratory conditions.

The continuous technological developments in DNA sequencing and assembly now make it affordable for individual research groups to acquire hundreds of complete bacterial genomes. Culture collections worldwide hold more than 1.5 million bacterial and fungal strains, large numbers of which are planned to be genome sequenced soon in several initiatives. Moreover, genomes can now be reconstructed from metagenomes thousands at a time (2) and massive metagenomic efforts such as the Earth Microbiome Project (3) plan to reconstruct around 500,000 genomes from diverse communities around the globe. It is not at all unrealistic to expect that within 5 to 10 years, the nucleotide sequence databases will contain millions of genome sequences of tens of thousands of biological species. Similarly, plant and fungal genome sequencing is also being scaled up, with the sequencing of thousands of eukaryotic genomes planned for the next few years. At the same time, complementary data are being gathered by using metabolomics, transcriptomics, and large-scale phenotyping studies. This presents tremendous opportunities for genome-based natural product discovery, as millions of BGCs can be scoured to identify high-value molecules and to predict and assess their functions in ecology.

**FIG 1:**
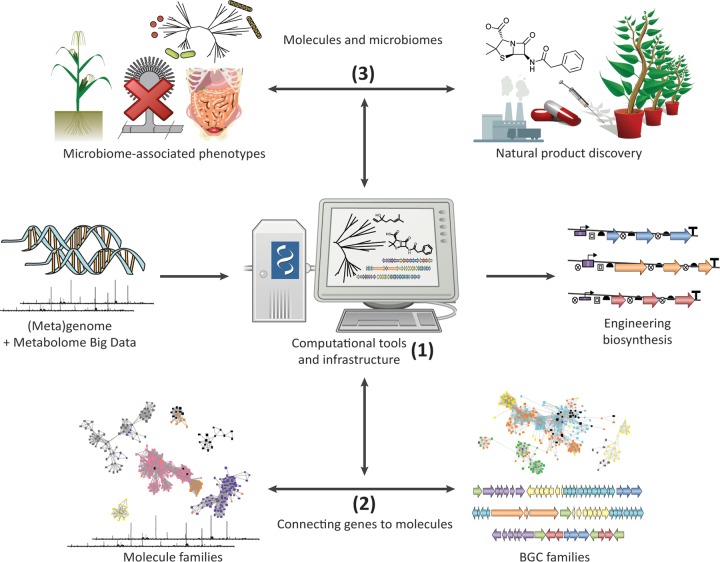
Overview of the research line of the Medema research group. We develop computational tools and infrastructure (part 1) to connect genes to molecules (part 2). With these technologies, we aim to accelerate natural product discovery and acquire an ecological understanding of the molecular mechanisms behind microbiome-associated phenotypes (MAPs) driven by specialized metabolism (part 3).

For the field studying specialized metabolite biosynthesis, this will require radical changes in the methods employed. Traditional approaches alone no longer suffice. Indeed, computation will play a more and more central role in the integration of large and diverse data sets and the generation of meaningful hypotheses for experimentation (Fig. 1).

## FROM INDIVIDUAL GENOMES TO PANGENOMES AND METAGENOMES

The starting point of natural product genome mining is the identification of BGCs. This procedure is fully automated by antiSMASH (4), a computational pipeline and web server that is currently jointly coordinated by the Medema group and the research group of Tilmann Weber at the Technical University of Denmark. AntiSMASH not only identifies BGCs, it also compares identified BGCs to experimentally characterized reference gene clusters from the MIBiG repository (1) and provides chemical structure prediction for several classes of natural products. Precomputed results are available online in the antiSMASH database (5). As an open-source project, antiSMASH is continuously extended with new functionalities by researchers worldwide through a model of open collaboration.

While antiSMASH effectively automates the analysis of individual genomes, it was already conceived in 2010 and therefore was never designed for the simultaneous exploration of hundreds or thousands of genomes or metagenomes. It is highly likely that the millions of BGCs that are becoming available will offer novel solutions for combating multidrug-resistant pathogens, treating cancer, and protecting crops against dangerous pathogens. The key challenge, however, is to find these much-desired needles in such a giant haystack.

To address this, we are currently developing novel solutions. To first acquire high-quality sets of BGCs from complex and large (meta)genomic data sets, new algorithms are being developed by us and several collaborating research groups to reconstruct full BGCs from metagenomic assemblies or from large sets of medium-quality draft genomes. Subsequently, the construction of BGC sequence similarity networks and the clustering of BGCs into gene cluster families (GCFs) are key methods to reduce the complexity of sets of thousands of BGCs and provide a bird’s-eye perspective on the underlying biosynthetic diversity (6–8). Our new software BiG-SCAPE (J. Navarro-Muñoz et al., unpublished data; https://git.wageningenur.nl/medema-group/BiG-SCAPE) streamlines and optimizes these methods to allow detailed analysis of the relationships between large numbers of BGCs without the need for supercomputing. Through annotation propagation with reference data from MIBiG (1), it allows rapid identification of GCFs with known and unknown functions and allows tracing of the taxonomic distribution of their pangenomic absence/presence patterns. Moreover, it provides a rich network visualization that allows interactive exploration of the data by dynamically browsing the network and searching it on the basis of taxonomic or Pfam identifiers. Finally, ongoing integration with CORASON (F. Barona-Gómez, personal communication; https://github.com/nselem/EvoDivMet) will facilitate phylogenetic reconstruction of GCFs to identify the relationships of the underlying BGCs at high resolution. In this way, scientists will be able to perform interactive exploration of biosynthetic diversity across, e.g., all genomes of the genus *Burkholderia*, multiple metagenomic data sets from plant rhizospheres, or all genomes associated with the human oral microbiome. Also, in the future, we plan to use precomputed BGC predictions for all publicly available genomes to populate an online database of standardized GCFs with curated annotations.

## CONNECTING GCFs TO MOLECULES

Exploration of biosynthetic diversity should never be a goal in itself, or it will remain nothing more than a “stamp-collecting exercise.” It should generate new hypotheses and illuminate real mechanisms and chemistry. Importantly, genomic data have the potential to greatly illuminate metabolomes. It has been estimated that in metabolomic data, >95% of the metabolite-derived masses cannot be linked to structures or functions (9). Matching of these masses to pathways and the strains producing these metabolites will play crucial roles in the identification of their structures and functions. Effective connection of genomic and metabolomic data will entail a bidirectional process, in which chemical features are predicted from BGCs, as well as from tandem mass spectrometric peak patterns.

The MIBiG initiative documented the connections between a large number of BGCs and the chemical structures of their products and allows for the standardized storage of data on enzymatic classes involved in these pathways, as well as their substrate specificities. This presents a rich data set to train algorithms to make powerful predictions about chemical (sub)structures of BGC products based on the DNA sequence of a BGC alone. For example, the SANDPUMA algorithm (10) for substrate specificity prediction of nonribosomal peptide synthetases made key improvements upon previous methods by extending earlier training sets with hundreds of new data points from MIBiG. Our recent work with the Dorrestein lab (11) showed that connecting such chemistry predictions to large-scale metabolomics of pseudomonads allowed the identification of new families of cyclic lipopeptides, which are known to mediate key interorganismal interactions in plant microbiomes.

The next challenge will be to take the prediction of substructures beyond peptides and include a wider variety of genetic features that can be correlated with mass spectrometric features. The former would include subclusters that encode pathways toward key precursors, enzyme-coding genes for group transfer of chemical monomers, and more “exotic” scaffold biosynthesis enzymes such as *trans*-acyltransferase polyketide synthases and terpene cyclases. This will make it possible to systematically extend metabologenomic matching (12) from correlation-based mapping alone to feature-based mapping. The emergence of more and more paired genomic-metabolomic data sets (with both types of data from the same samples) will undoubtedly accelerate these efforts.

## CONNECTING MICROBES TO PLANT AND HUMAN HOSTS

Even molecules are not the endpoint of natural product discovery. In the end, it is their function that matters most. Such function goes beyond pharmacological activities such as antibiotic or cytotoxic activity, and from a fundamental perspective, is really about the fitness effects of molecules on both the producing organism and members of the community surrounding it. We feel that microbiomes and their chemical language should be approached from a systems perspective. In the end, this allows acquiring a real understanding of how specialized metabolites and other molecular mechanisms shape key microbiome-associated phenotypes (MAPs) (15), such as disease suppression or growth promotion, in plant and human microbiomes. These interactions comprise both host and microbial components.

From the microbial side, metagenomics and metatranscriptomics allow the identification of differential abundance and differential expression of BGCs in microbial communities, which can be correlated with MAPs. Again, the grouping of BGCs into GCFs that represent functional traits across multiple organisms plays a key role here, as they allow metagenome-wide association studies of these genetic traits with (un)desirable MAPs. This then generates hypotheses that can be tested in the laboratory through the isolation and characterization of specific strains (e.g., in synthetic microbial communities) or through heterologous expression of BGCs refactored through synthetic biology.

From the host side, it is increasingly appreciated that small molecules also play key roles in shaping the microbiota. In plants, this includes root exudation (positive regulation), as well as secretion of defense compounds (negative regulation). The technological ability to sequence high-quality plant genomes along with (time series) transcriptomes across a range of conditions paves the way for the development of genome mining strategies for the identification of plant biosynthetic pathways by studying patterns of genomic colocalization, coexpression, and coevolution of enzyme-coding genes, in combination with metabolomic and phenotypic data (13). Our recently launched platform plantiSMASH, the plant equivalent of antiSMASH, facilitates many of these analyses. In the human microbiome, chemical interactions between hosts and microbes are also of key importance. For example, gut microbes transform bile acids produced by the liver into a wide range of secondary bile acids, many of which have a major impact on human health (14). Building an “antiSMASH”-like platform for the identification of microbial pathways involved in such chemical transformations has great potential to foster our understanding of the role of the human and animal microbiota in health and disease.

Concluding, we are excited about the prospects for the reinvigorated study of specialized metabolism and are convinced that the integration of cutting-edge omics technologies, computation, and foundational chemical and ecological concepts will provide many new insights into the chemical language of life and its many biotechnological applications that can improve human well-being.
